# Gas-Phase Oxidation of Atmospherically Relevant Unsaturated Hydrocarbons by Acyl Peroxy Radicals

**DOI:** 10.1021/jacs.4c02523

**Published:** 2024-05-07

**Authors:** Dominika Pasik, Benjamin N. Frandsen, Melissa Meder, Siddharth Iyer, Theo Kurtén, Nanna Myllys

**Affiliations:** † Department of Chemistry, University of Helsinki, Helsinki 00014, Finland; ‡ Institute for Atmospheric and Earth System Research, University of Helsinki, Helsinki 00014, Finland; § Aerosol Physics Laboratory, Tampere University, Tampere 33014, Finland

## Abstract

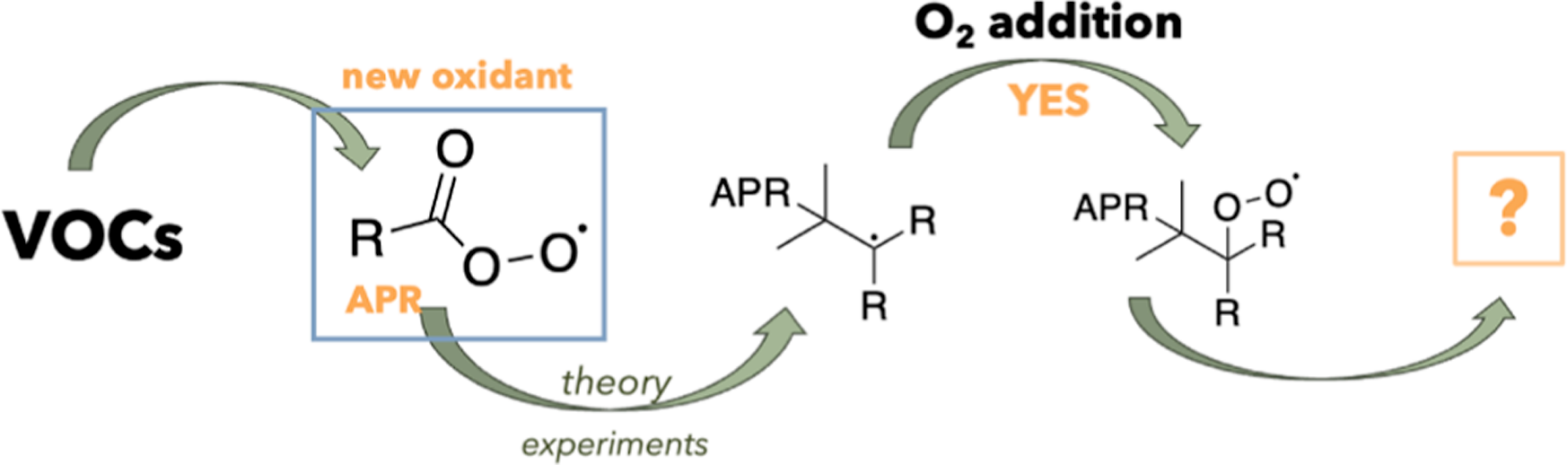

This study assesses the atmospheric impact of reactions between unsaturated hydrocarbons such as isoprene and monoterpenes and peroxy radicals containing various functional groups. We find that reactions between alkenes and acyl peroxy radicals have reaction rates high enough to be feasible in the atmosphere and lead to high molar mass accretion products. Moreover, the reaction between unsaturated hydrocarbons and acyl peroxy radicals leads to an alkyl radical, to which molecular oxygen rapidly adds. This finding is confirmed by both theoretical calculations and experiments. The formed perester peroxy radical may either undergo further H-shift reactions or react bimolecularly. The multifunctional oxygenated compounds formed through acyl peroxy radical + alkene reactions are potentially important contributors to particle formation and growth. Thus, acyl peroxy radical-initiated oxidation chemistry may need to be included in atmospheric models.

## 1 Introduction

Atmospheric volatile organic compounds (VOCs) are directly emitted from anthropogenic and biogenic sources into the atmosphere, playing an important role in the chemistry of aerosols and the global carbon cycle.^1−3^ A reaction class of particular interest is the subset of VOC oxidation pathways leading to (extremely) low-volatility organic compounds [(E)LVOCs] which can participate in the formation and growth of aerosol particles.^4,5^ Isoprene and monoterpenes are two major representatives of VOCs from biological sources. Their high reactivity plays a crucial role in the photochemical reactions occurring within the troposphere, thereby influencing the levels of secondary air pollutants such as secondary organic aerosol.^6^

The main oxidants in the atmosphere are ozone (O_3_), hydroxyl radicals (OH^•^), and nitrate radicals (NO_3_^•^), and the initial steps of their reactions with common atmospheric VOCs have been extensively investigated.^7−9^ Indeed, recent studies have led to the critical discovery that intramolecular hydrogen shifts of peroxy radicals, followed by rapid addition of molecular oxygen, i.e., autoxidation mechanisms, can occur for some peroxy radical intermediates under atmospheric conditions at rates competitive with bimolecular radical reactions.^10,11^ These reactions can rapidly lead to highly functionalized and often low-volatility aerosol precursors. However, unimolecular reactions of peroxy radicals compete with bimolecular reactions with other radicals such as HO_2_, NO_*x*_, and ROO^•^, that lead primarily to radical termination. This limits the atmospheric average lifetime of ROO^•^ with respect to bimolecular pathways from seconds to minutes. Therefore, ROO^•^ H-shifts should have unimolecular rates on the order of at least 0.01 s^–1^ to be competitive.

Formation of closed-shell dimeric accretion products such as organic peroxides and esters from recombination reactions of peroxy radicals has been the focus of multiple computational and experimental studies.^12−15^ These recombination reactions are believed to inevitably lead to tetroxide RO_4_R intermediates, which then decompose into (triplet) molecular oxygen and ^3^(RO···R′O) intermediate complexes. Organic peroxide (ROOR) formation occurs via intersystem crossing of this complex from the triplet to the singlet surface.^15,16^ Formation of esters [R(O)OR] similarly occurs via β-scisson of R(O)CO alkoxy radicals inside triplet ^3^(RO···R′O) intermediate complexes, and subsequent alkoxy–alkyl recombination, likely involving intersystem crossing.^12^ Autoxidation processes combined with dimerization reactions can rapidly produce low-volatility compounds, which in turn participate in particle formation and growth processes. In addition to regular peroxy radicals, the atmosphere contains on the order of 10^7^–10^8^ cm^–3^ of short-chain acyl peroxy radicals [R(O)OO^•^, APR].^17,18^ Moreover, APRs are known to be more reactive than ROO^•^.^19,20^ Bimolecular APR reactions with NO_2_, producing long-lived acyl peroxy nitrates, have been extensively studied.^21,22^ Unimolecular H-shift reactions of acyl peroxy radicals have been found to have rate constants in the range of from 10^–8^ to 10^5^ s^–1^, with larger and more functional radicals tending to have faster reaction rates.^23,24^ Additionally, R(O)OO^•^ + R(O)OO^•^ dimerization reactions are suggested to lead to low-volatility products through a similar mechanism as the ROO^•^ + ROO^•^ reaction.^25,26^ As APRs have higher reactivities than other peroxy radicals, different mechanisms might control their atmospheric chemistry. Specifically, APRs may be able to react directly with closed-shell species such as alkenes, opening up new potential pathways for accretion product formation.

The reactions of ROO^•^ with VOCs used to be considered negligible at room temperature and, thus, were disregarded in atmospheric chemistry. This presumption was solely grounded in combustion studies, where epoxide formation is the dominant pathway and the extrapolated rate constant for that reaction was found to be very low.^27^ Recently, Nozière et al. (2023) demonstrated that unsaturated hydrocarbons indeed react with some peroxy radicals, but epoxide formation cannot be the primary pathway at room temperature.^28^ Instead, they proposed that the reaction between ROO^•^ and the alkene proceeds through an accretion pathway. Therefore, it is crucial to thoroughly investigate these reactions as alkene reactions with functionalized peroxy radicals could be competitive, potentially serving as additional sinks for atmospheric unsaturated hydrocarbons.

The aim of this study is to assess the atmospheric relevance of reactions involving unsaturated hydrocarbons and various functional peroxy radicals by determining their bimolecular reaction rate coefficients and formation rates under atmospheric conditions. These reactions hold potential atmospheric significance, as the resulting products contain a carbon radical center to which molecular oxygen can add, potentially allowing further reactions such as autoxidation and dimerization, and thus leading to compounds with low saturation vapor pressure.

## 2 Methods

### 2.1 Computational Details

In our previous study, we introduced a cost-effective and computationally efficient methodology for investigating accretion reactions between unsaturated hydrocarbons and organic peroxy radicals.^29^ Transition states (TS) of the accretion reaction were located from the relaxed potential energy surface scan using density functional theory (DFT) ωB97X-D/6-31+G* level.^30−32^ To search the conformational space, we used the CREST program, which serves as a tool for semiempirical calculations and performs conformational sampling at the extended tight-binding level GFN-xTB. The obtained TS serves as an input in the CREST configurational search, which occurs by freezing the reactive area of the TS structure (i.e., the distance for forming the C–O bond).^33,34^ In the next step, the generated conformers for TS-structures were optimized at the DFT level while keeping the C–O bond distance frozen. Subsequently, the full TS state optimization and frequency calculation were performed. TS structures were verified by the presence of exactly one imaginary frequency. Additionally, conformers optimizing to the same geometry (duplicates) have been removed based on electronic energy, dipole moment, and squared rotational constant. On top of the DFT structures, linear-scaling coupled-cluster with single and double excitations as well as perturbative-inclusion-of-triples single-point calculations were performed using the DLPNO–CCSD(T)/aug-cc-pVTZ to obtain more accurate electronic energy values.^35^ For all conformers, the zero-point corrected energies were calculated. Barrier heights were computed as a difference between TS and reactants energies. All DFT calculations were conducted using Gaussian 16 RevC.02 software,^36^ and single-point energies were calculated using ORCA version 5.0.3.^37,38^

The reaction rate coefficients were calculated using the multiconformer transition state theory (MC-TST) approach for bimolecular reaction following expression^39,40^
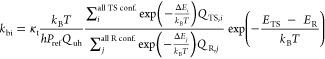
1where κ_t_ is the quantum-mechanical tunneling coefficient (κ_t_ = 1 is used for the reaction between atoms other than hydrogen), *T* is the temperature (=298.15 K), *h* is the Planck’s constant, *P*_ref_ is the reference pressure (=2.45 × 10^19^ cm^–3^), and *k*_B_ is the Boltzmann’s constant. *Q*_R,*j*_ and *Q*_TS,*i*_ are the partition functions of the reactant (peroxy radical) and transition state conformers, respectively, *Q*_uh_ is a partition function for unsaturated hydrocarbons where only the lowest energy conformer was included in the partition function due to the large energy gap between that and the higher energy conformers. Δ*E*_*j*_ and Δ*E*_*i*_ correspond to the zero-point corrected electronic energies of the reactant and transition state conformers relative to the lowest energy conformers, respectively. *E*_R_ and *E*_TS_ are the zero-point corrected electronic energies of the lowest energy reactant and transition state conformers. Partition functions were calculated at the ωB97X-D/6-31+G* level of theory, while the reaction barrier (*E*_TS_ – *E*_R_) includes the DLPNO–CCSD(T)/aug-cc-pVTZ energy correction.

For the hydrogen shift reactions, the unimolecular reaction rate coefficients were calculated following the equation^41,42^
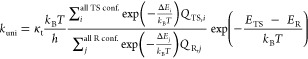
2

Additionally, intrinsic reaction coordinate calculations for connecting the correct reacting conformers were performed.

To calculate the tunneling coefficient, κ_t_, we employed the Eckart tunneling method. This method involves a one-dimensional calculation where the tunneling coefficient is determined by solving the 3-parameter equation for an asymmetrical one-dimensional potential, known as the Eckart potential. The calculation requires the energy of the transition state, as well as the energy of the reactant and product, which is connected to the lowest TS structure, as well as the frequency of the imaginary vibrational mode corresponding to the reaction. The Eckart tunneling method has been previously successfully used for calculating hydrogen shift reaction rates for similar reactions.^43−45^

### 2.2 Experimental Details

The ozonolysis products of 2,3-dimethyl-2-butene (tetramethylethylene, TME, Sigma-Aldrich ≥99%) were studied using chemical ionization mass spectrometry (CIMS). We reacted TME with ozone in a glass flow reactor and monitored the ozonolysis products with a chemical ionization orbitrap mass spectrometer (CI-orbitrap; Q Exactive Plus Orbitrap MS, Thermo Fisher Scientific Inc.). Riva et al. (2019) showed that the CI-orbitrap can be used to detect oxidized species,^46^ and the sensitivity of the instrument toward these species was optimized by Cai et al. (2022).^47^ The CI-orbitrap consists of a chemical ionization inlet (CI-inlet), where the sample molecules are ionized using reagent ions produced using X-ray.^48^ Typically, nitrate (NO_3_^–^) CIMS is used when studying highly oxygenated organic molecules as nitrate is highly selective toward the higher oxidized species.^11,49^ Nevertheless, CIMS can be used to monitor also less oxidized products by using aminium ions.^46,50^ In this study, we used *N*-butylamine to produce *N*-butylammonium [C_4_H_12_N^+^, *N*-butylaminium (NBA)] reagent ions and monitor the oxidation products with at least one oxygen atom.

**Figure 1:**
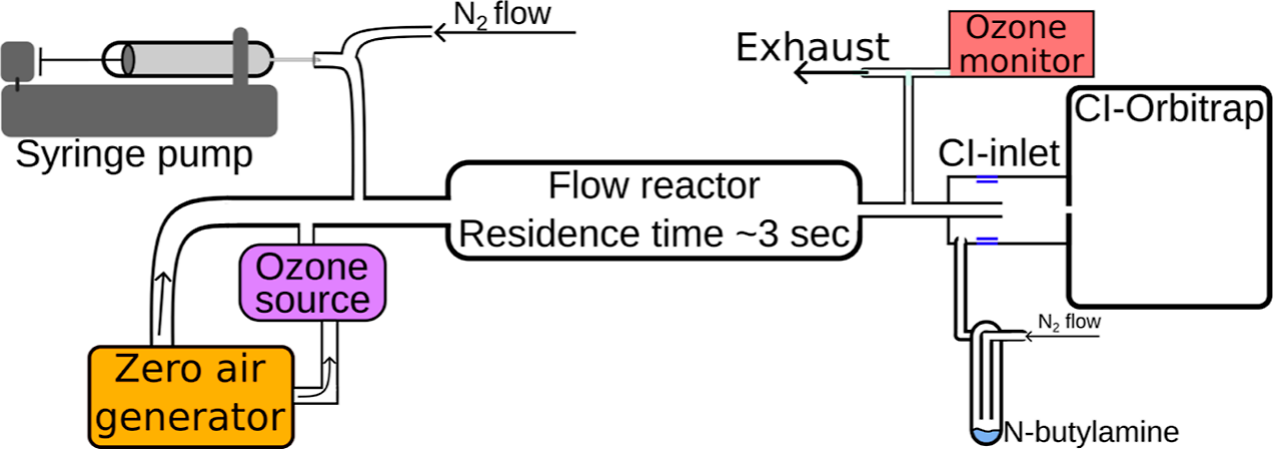
Schematic of the experimental setup.

Figure 1 contains an illustrative overview of the experimental setup. The experiments were carried out in positive ion mode utilizing NBA as the reagent ion. The temperature was 296 ± 2 K. Liquid *N*-butylamine (Sigma-Aldrich, ≥99.5%) was placed in a glass bubbler but with the liquid level below the exhaust tip, such that it was not bubbling. A 30 mL min^–1^ N_2_ flow through the bubbler was used to carry *N*-butylamine into the CI-inlet, where it was ionized using two X-ray sources prior to introducing the ions to the sample flow, see Cai et al. (2022) for a detailed CI-inlet description.^47^ Ozone was generated from zero air using an ozone generator (Dasibi 1008-PC ozone generator). Ozone concentration was monitored using a UV absorbance ozone monitor (model 400, Teledyne instruments) and kept at 131 ± 1 ppb. The total flow rate through the flow reactor was set to 13.2 ± 0.2 L min^–1^, and the sample flow rate to the CI-orbitrap was 10.0 ± 0.2 L min^–1^. The flow reactor had an estimated residence time of 3 s. Liquid TME was injected into the flow reactor by using a syringe pump for a controlled injection rate using an N_2_ carrier gas flow. We used 0.5, 1.0, and 2.0 μL/h injection rates which correspond to initial TME concentrations of 128, 256, and 512 ppb prior to mixing with ozone, respectively, see the Supporting Information for concentration calculation details. The experimental procedure involved carrying out two control experiments, one with the ozone source turned on without TME injection and one with TME injection but without ozone. Measurement settings were optimized for sensitivity as described by Cai et al. (2022), namely using averages of 100 microscans per recorded mass spectrum.^47^

To clarify the steps taken during each experiment, we describe the experimental procedure next. First, we conducted a background measurement with the ozone generator and all flows turned on, and the TME syringe was placed ready and connected to the experiment, but with the syringe pump turned off. Initially, when the TME syringe was inserted into the septum and placed in the syringe pump, some amount of TME would evaporate from the needle headspace and into the flow reactor. Background measurements were done after this TME had evaporated, and its products were no longer detectable by the CI-orbitrap. Next, we conducted the three TME ozonolysis measurements by turning on the syringe pump, starting with the 0.5 μL/h TME injection rate. For each TME injection rate, a series of data points were acquired after letting the signals stabilize. Last, after the final data point was recorded for the 2.0 μL/h TME injection rate measurement, the ozone generator was turned off and the TME injection rate was kept at 2.0 μL/h to conduct the ozone-free background measurement. At the end of the full series of measurements, the syringe was removed and cleaned while the flow reactor was baked-out at 200 °C.

The CI-orbitrap data was analyzed using Orbitool^51^ and Matlab R2023b. All signal intensities were normalized relative to the sum of the NBA and NBA dimer signal.

## 3 Results and Discussion

### 3.1 Isoprene + Functional Peroxy Radicals

**Figure 2:**
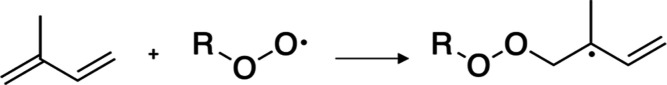
Studied pathway for accretion reaction between isoprene and peroxy radicals (ROO^•^) leading to an allylic radical with tertiary/primary radical sites.

Recently, Pasik et al. (2024) demonstrated that the reaction between isoprene and aliphatic peroxy radicals (methyl, ethyl, propyl peroxy radicals) is hindered by high energy barriers.^29^ The calculated rate coefficients (on the order of 10^–21^ cm^3^ s^–1^) suggest the insignificance of these reactions in an atmospheric context. As expected from other radical addition mechanisms, the most favorable and lowest-barrier pathway for ROO^•^ addition to isoprene corresponded to the formation of tertiary alkyl radicals, which are resonance stabilized. Consequently, in this study, we explore this reaction pathway for isoprene with acyl peroxy radicals, as well as peroxy radicals with other functional groups, to evaluate their reactivity with isoprene (see Figure 2).

We selected a diverse set of functional peroxy radicals. Table 1 shows that for acyl peroxy radicals (RC(O)OO^•^, APR), the calculated electronic energy barriers are remarkably low (<1 kcal/mol). However, for the other functional peroxy radicals, the energy barriers are too high (3–7 kcal/mol) for these reactions to be competitive, making them insignificant for atmospheric chemistry. Interestingly, for the β-oxo system with the C=O group shifted one carbon further from the peroxy group [CH_3_C(O)CH_2_OO^•^ in comparison to C_2_H_5_C(O)OO^•^], the calculated energy barrier value is significantly higher. Therefore, acyl peroxy radicals appear to be much more reactive toward double bonds than other peroxy radicals. Population analysis from atomic polar tensor (APT) showed that terminal oxygen in CH_3_C(O)OO^•^ has less negative charge compared to CH_3_C(O)CH_2_OO^•^ or CH_3_CH_2_OO^•^ making it more reactive toward double bonds (see Figure S3 in Supporting Information). This is also consistent with theoretical studies by Hasson et al. (2005).^52^ The detailed reasons for the increased reactivity should be confirmed through methods delving into the electronic structure of these radicals, which is outside the scope of this study. Thus, we will focus only on APR chemistry in this paper.

**Table 1:** Zero-Point Energy Corrected Barrier Heights (Δ*E*^TS^ [kcal/mol]), Gibbs Free Energy Barriers (Δ*G*^TS^ [kcal/mol]) and MC-TST Bimolecular Reaction Rate Coefficients at 298 K [*k*_bi_ (cm^3^ s^–1^)] for Studied Reactions Calculated at the DLPNO–CCSD(T)/aug-cc-pVTZ//ωB97X-D/6-31+G* Level of Theory^a^

radical	Δ*E*^TS^	Δ*G*^TS^	*k*_bi_
**HC(O)OO**^•^	0.0	11.0	4.6 × 10^–15^
**CH**_**3**_**C(O)OO**^•^	0.7	11.5	5.3 × 10^–16^
**NH**_**2**_**C(O)OO**^•^	0.8	12.3	6.3 × 10^–16^
CH_3_SCH_2_OO^•^	7.2	19.5	2.5 × 10^–20^
NH_2_CH_2_OO^•^	7.4	18.9	8.9 × 10^–21^
C_6_H_5_CH_2_OO^•^	6.7	18.8	5.0 × 10^–21^
**C**_**6**_**H**_**5**_**C(O)OO**^•^	0.1	11.6	1.8 × 10^–15^
**C**_**2**_**H**_**5**_**C(O)OO**^•^	–0.3	11.9	1.7 × 10^–15^
CH_3_C(O)CH_2_OO^•^	3.4	15.4	8.0 × 10^–18^

a APRs are bolded.

It is worth noting that this study focuses on short-chain aliphatic acyl peroxy radicals (with up to 3 carbons), as these are likely to have longer lifetimes with respect to unimolecular reactions and hence higher atmospheric concentrations. Multiple studies have demonstrated that long-chain APRs rapidly undergo hydrogen shift and further autoxidation reactions leading to the transformation of APR into peroxy radicals.^10,20,23^ These reactions result in a decrease in concentrations of longer-chain APRs, and thus also in the yields of their reactions with unsaturated hydrocarbons. However, note that benzoyl peroxy radicals [C_6_H_5_C(O)OO^•^] can be expected to be too rigid for intermolecular H-shifts and thus potential participants for bimolecular reactions.

Knowing the approximate atmospheric concentrations of the reactants, it is possible to calculate the reaction rate (i.e., the rate of formation of products) for a given chemical reaction. The only reported atmospheric APR concentration that we could find is for CH_3_C(O)OO^•^ with a value of 1.5 × 10^8^ cm^–3^.^18^ This value is from a Villenave et al. (1998) study, which corresponds to the concentration of APRs in hydrocarbon-rich remote atmospheres in low-NO_*x*_ conditions. It is worth noting that measuring the concentration of APR is challenging, and to the best of our knowledge, there are only few studies reporting atmospheric concentrations of APRs. Therefore, it is important to keep in mind that the given value carries some degree of uncertainty. For isoprene, a representative continental boundary-layer concentration is 10^11^ cm^–3^.^18,53^

A reasonable global average for the lower-troposphere concentration of OH^•^ is 10^6^ cm^–3^.^7^ As the isoprene concentration is much larger than the radical concentration, we will use pseudo-first order reaction rates to discuss atmospheric relevance of APR-initiated oxidation. The primary sink for isoprene is its reaction with OH^•^, which has a pseudo-first order reaction rate on the order of 10^–4^ cm^3^ s^–1^ (*k*_bi_ = 10^–10^ cm^3^ s^–1^,^54^ rate = *k*_bi_[VOC][OH] = *k*_pseudo_[OH]).

For the APR-initiated isoprene oxidation reaction, the pseudo-first order reaction rate is on the order of 10^–7^ cm^3^ s^–1^. That would correspond to around 0.1% of the OH-initiated oxidation rate leading to minor direct atmospheric impact.

On the other hand, it leads to the formation of products with higher molar mass and larger number of oxygen atoms, so despite its small overall yield, it may contribute to the production of extremely low volatility compounds. This reaction is also likely more significant in high-concentration experiments and should thus be taken into account when analyzing the results. Additionally, it is noteworthy that the reaction rate calculations for APR-initiated isoprene oxidation only account for the reaction shown in Figure 2, but the OH-initiated isoprene oxidation measurements correspond to the total OH addition reaction rate for four possible positions.

### 3.2 APR-Initiated Oxidation

**Figure 3:**
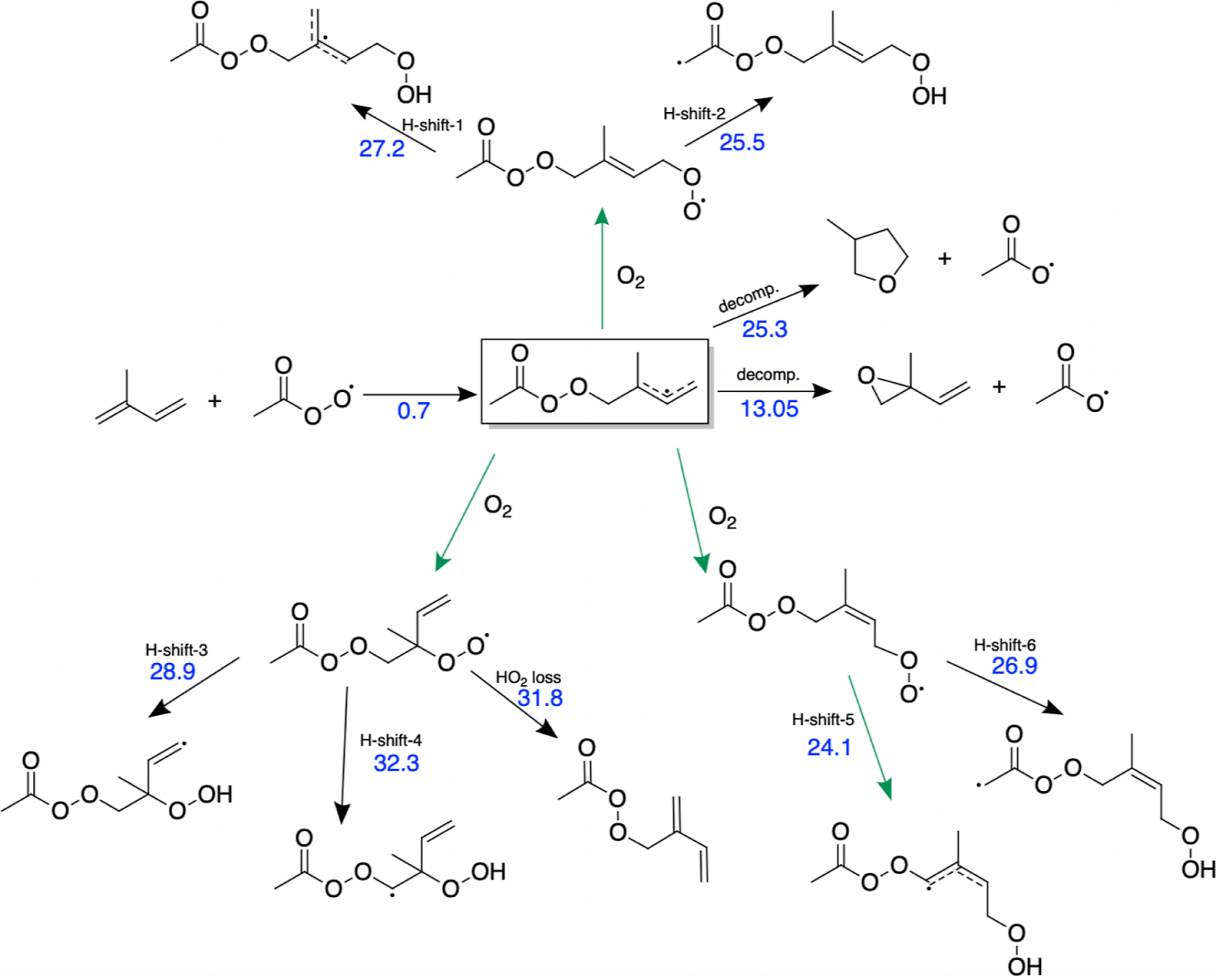
Studied pathway for the accretion reaction between isoprene and acetyl peroxy radical and subsequent oxidation reaction channels. Energy barrier heights [kcal/mol] are marked with the blue color and the dominant pathway is following the green labeling. Please, note that product of H-shift-4 reaction is not a distinct minimum and follows the decomposition pathway (see Figure 4).

As shown in the previous section, the reaction between isoprene and acyl peroxy radicals exhibits low energy barriers and high reaction rate coefficients, making these reactions competitive with other isoprene loss channels under atmospheric conditions. To study subsequent reactions, we focus on the isoprene + CH_3_C(O)OO^•^ acetyl peroxy radical system and investigate the possible reaction pathways (see Figure 3). The direct accretion product is a perester alkyl radical, to which molecular oxygen can rapidly add. An alternative pathway leads the perester alkyl radical to decompose into an epoxide and alkoxy radical. This pathway is known to be dominant under combustion temperatures, and it has also been suggested to occur under room temperature.^19,27,55^ After O_2_ addition, the formed perester peroxy radical could undergo a series of hydrogen shifts and oxygen additions, i.e., autoxidation, or react via bimolecular pathways. Depending on their structures, the QOOH radicals formed by hydrogen shifts can also undergo dissociation or HO_2_ loss reactions. We have calculated the energy barriers for the reaction pathways that the isoprene + acetyl peroxy radical product could possibly undergo and determined the reaction rate coefficients using the unimolecular MC-TST method (eq 2). The lowest barrier reactions are illustrated in Figure 3. Information about other studied pathways can be found in the Supporting Information (Figure S1, Table S1).

The calculations show that the decomposition of the isoprene + CH_3_C(O)OO^•^ accretion product by three-membered ring epoxide formation has an energy barrier of 13 kcal/mol, while the addition of oxygen has a negative barrier. A typical reaction rate coefficient value for the addition of O_2_ to a resonantly stabilized alkyl radicals is 6 × 10^–13^ cm^3^ s^–1^,^56^ while our calculated decomposition reaction rate coefficient is 9 × 10^2^ s^–1^. Assuming an atmospheric O_2_ concentration of 10^19^ cm^–3^, the rate for the addition of the O_2_ is 4–5 orders of magnitude higher, indicating that this reaction can be considered the dominant pathway at room temperature. The molecular oxygen addition reaction leads to the formation of a perester peroxy radical, which can then undergo either bimolecular reactions, unimolecular H-shifts, or concerted HO_2_ loss. It is noteworthy that the epoxidation of perester alkyl radical is known to occur in combustion processes,^27^ however, a kinetic study by Nozière et al. (2023) showed that epoxy formation channel cannot be the main pathway at room temperature.^28^

**Figure 4:**
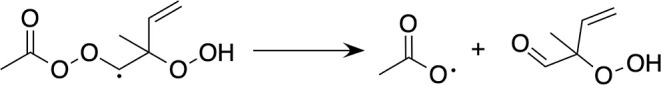
Decomposition pathway of H-shift-4 reaction product.

Table 2 shows that the lowest energy barrier and highest reaction rate coefficient are observed for the H-shift labeled H-shift-5. However, the thermodynamically most favorable pathway would be H-shift-4, as it leads to prompt decomposition of formed alkyl radical (see Figure 4), and subsequently products with remarkably lower energy than the reactant perester peroxy radical (−27.7 kcal/mol).^57^

**Table 2:** Zero-Point Energy Corrected Barrier Heights [kcal/mol] and Unimolecular MC-TST Reaction Rate Coefficients at 298 K (*k*_uni_ [s^–1^]) for Studied H-Shift Reactions Calculated at the DLPNO–CCSD(T)/aug-cc-pVTZ//ωB97X-D/6-31+G* Level of Theory

reaction	Δ*E*^TS^	*k*_uni_
H-shift-1	27.2	2.6 × 10^–5^
H-shift-2	25.5	8.4 × 10^–7^
H-shift-3	28.9	4.9 × 10^–9^
H-shift-4	32.3	3.7 × 10^–8^
H-shift-5	24.1	1.4 × 10^–3^
H-shift-6	26.9	2.5 × 10^–7^

This is not observed for the remaining pathways, for which the products have similar or higher energies than the reactant. The closed-shell product of H-shift-4 is a hydroperoxy aldehyde (HPALD), a well-known class of isoprene oxidation products.^7^ The product of the H-shift-5 reaction has a resonance stabilized alkyl radical center adjacent to the peroxide group. It may thus undergo decomposition reactions, also resulting in an HPALD compound. Our findings, indicating that the decomposition is rapid despite the resonance stabilization, are consistent with multiple studies demonstrating that α-QOOR radicals are not stable and undergo decomposition.^57−59^ However, we were able to optimize the reaction product as a distinct minimum on the potential energy surface, likely due to the resonance stabilization of the delocalized double bond. Frequency analysis confirmed that the product is a minimum structure. If the lifetime with respect to the decomposition reaction is long enough to permit O_2_ addition, this would represent a case of APR-initiated autoxidation. The loss of HO_2_ from one of the RO_2_ radicals was examined, as depicted in Figure 3. This process includes abstraction of hydrogen from the methyl group of the isoprene unit (see Figure S2). The calculations show that while concerted HO_2_ loss has a lower barrier than stepwise HO_2_ loss, even the concerted loss barrier is fairly high, and thus HO_2_ loss would not compete with bimolecular reactions, or even with the unimolecular H-shifts. However, all of the H-shifts illustrated in Figure 3 are too slow to be competitive with bimolecular ROO^•^ reactions, even under the cleanest conditions.

### 3.3 Reactivity of Unsaturated Hydrocarbons

**Figure 5:**
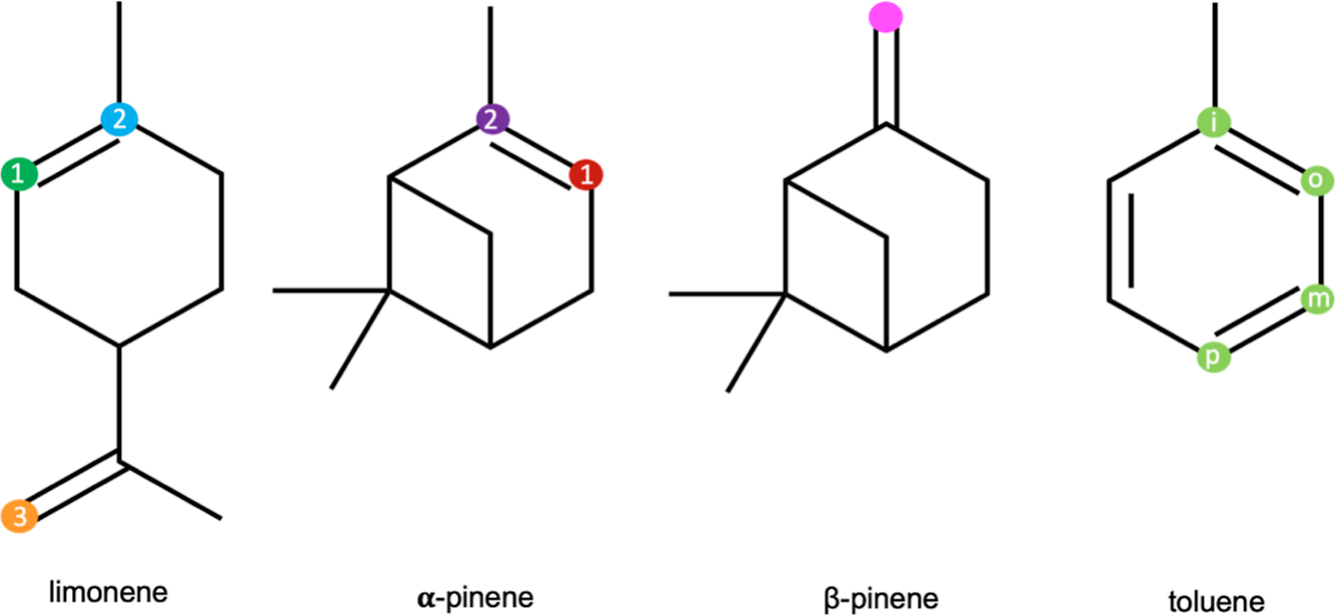
Structures of hydrocarbons undergoing the accretion reaction with APR. The considered addition positions are marked with colorful dots.

As the reaction between isoprene and acyl peroxy radicals demonstrates low barriers and relatively high reaction rate coefficients, we conducted analogous calculations for other atmospheric-relevant compounds such as limonene, α-pinene, β-pinene, and toluene. For these compounds, the addition of the CH_3_C(O)OO^•^ radical to the double bond was considered at multiple positions, excluding additions that would result in the formation of a primary alkyl radical for β-pinene and limonene. Figure 5 schematically represents the considered sites of addition of APR to an unsaturated hydrocarbon. The obtained reaction barriers as well as the calculated reaction rate coefficients are presented in Table 3. The labeling in Figure 5 corresponds to the labeling in Table 3.

**Table 3:** Zero-Point Energy Corrected Barrier Heights (Δ*E*^TS^ [kcal/mol]), Gibbs Free Energy Barriers (Δ*G*^TS^ [kcal/mol]), Bimolecular MC-TST Reaction Rate Coefficients at 298 K (*k*_bi_ [cm^3^ s^–1^]), and Pseudo First-Order Reaction Rates at 298 K (*k*_pseudo_[radical] [cm^3^ s^–1^]) for Studied Accretion Reactions of Monoterpenes and Toluene with Acetyl Peroxy Radical Calculated at the DLPNO–CCSD(T)/aug-cc-pVTZ//ωB97X-D/6-31+G* Level of Theory^a^

system	Δ*E*^TS^	Δ*G*^TS^	*k*_bi_	*k*_pseudo_[APR]	*k*_pseudo_[OH]
limonene-1	0.1	11	9.5 × 10^–15^	1.4 × 10^–6^	2.0 × 10^–4^^61^
limonene-2	3.2	15.1	4.3 × 10^–18^		
limonene-3	1.7	13.3	5.3 × 10^–17^		
α-pinene-1	3.7	14.7	3.5 × 10^–18^	6.8 × 10^–9^	5.6 × 10^–5^^62^
α-pinene-2	2.0	13.0	4.2 × 10^–17^		
β-pinene	1.3	12.4	2.7 × 10^–17^	4.0 × 10^–9^	8.8 × 10^–5^^62^
tolunene-i	5.2	18.1	6.0 × 10^–21^	5.9 × 10^–12^	6.0 × 10^–6^^63^
tolunene-*o*	5.1	16.6	2.5 × 10^–20^		
tolunene-*m*	6.7	19.1	4.6 × 10^–21^		
tolunene-*p*	6.6	18.5	3.7 × 10^–21^		

a Concentrations of reactants used to calculate reaction rates: [CH_3_C(O)OO^•^] = 1.5 × 10^8^ cm^–3^, [OH] = 1.0 × 10^6^ cm^–3^.

In the case of limonene, APR addition is possible for four different carbon atoms with double bonds. However, we only considered three possibilities, as based on previous results for the reaction of isoprene + ROO^•^ and relying on chemical intuition, it can be determined that addition resulting in the formation of a primary radical will not play a significant role. The data presented in Table 3 indicates that the most probable scenario is APR addition to the double bond in the cyclic ring, forming a stabilized tertiary radical structure. Nonetheless, the other two reactions also exhibit competitive energy barriers. Given the relatively high concentrations of limonene (2.7 × 10^10^ cm^–3^) and APR (CH_3_C(O)OO^•^, 1.5 × 10^8^ cm^–3^) in the atmosphere, the calculated total pseudo first-order reaction rate is 1.4 × 10^–6^ cm^3^ s^–1^. These values are sufficient to consider these reactions feasible as minor but not necessarily negligible sources of accretion products under atmospheric conditions. The reaction of limonene with OH^•^ in the atmosphere proceeds with a pseudo-first order reaction rate of 2 × 10^–4^ cm^3^ s^–1^.^60^ Thus, the reaction with APR accounts for up to 1% of the limonene sink. This helps explain why the suggested pathway has not been discovered earlier, for example, in field or chamber studies.

Interesting observations are made for α-pinene, as calculations show a lower energy barrier for the addition reaction, which leads to the formation of a secondary alkyl radical (position 2). Addition to the alternative position (position 1), although it leads to the formation of a tertiary alkyl radical, shows a higher energy barrier. This may be due to the presence of a steric hindrance in the TS for adding APR to the position 2 that is missing in the TS for position 1. The total pseudo first-order reaction rate of α-pinene with APR being 6.8 × 10^–9^ cm^3^ s^–1^, is still lower compared to the reaction rate with OH^•^ radical, which is in order of 10^–5^ cm^3^ s^–1^. Thus, APR-initiated oxidation constitutes less than 0.1% of the total α-pinene sink.

For β-pinene, we considered the addition to the double bond, leading to the formation of a tertiary radical. The obtained energy barrier is small (1.3 kcal/mol), and the calculated reaction rate coefficient is of a similar order of magnitude as the reaction rate coefficients for α-pinene. The concentrations of α- and β-pinene in the atmosphere are of similar value. As a result, the considered reaction with APR yields a pseudo first-order reaction rate of 10^–9^ s^–1^. Similar to α-pinene, this value is lower than that of the competing reaction with the OH^•^ radical (10^–5^ cm^3^ s^–1^).

Toluene is the most abundant alkylbenzene in the atmosphere. Numerous studies indicate that the main pathway for the removal of toluene is its addition reaction with OH^•^ accounting for around 93% in total, with bimolecular reaction rate coefficient *k*_OH_ = 6 × 10^–11^ cm^3^ s^–1^, but also the H-abstraction from the methyl group is possible accounting for around 7%.^63−65^ We investigated whether the reaction with APR could also be one of the sinks for toluene in the atmosphere. From the four possible positions for additions we considered, we found the APR addition to the ortho position to be the most preferential. This is consistent with the results obtained by Suh et al. (2002),^66^ where they considered the addition reactions of OH^•^ radical to toluene. However, even for the most preferred position, the addition of APR to toluene occurs through a fairly high barrier (5 kcal/mol), resulting in a low bimolecular reaction rate coefficient (3 × 10^–20^ cm^3^ s^–1^). For these reasons, we would not consider this or other APR + aromatic reactions significant for atmospheric chemistry.

### 3.4 Flow Reactor TME Ozonolysis Results

The ozonolysis of TME involves the formation of acetone and the (CH_3_)_2_COO Criegee intermediate, which undergoes unimolecular decomposition to form OH^•^ radicals and acetonyl. Acetonyl then rapidly adds O_2_ to form acetonyldioxyl.^67^ Given the relatively high TME concentrations employed in the experiments, a nearly equal amount of 2-hydroxy-1,1,2-trimethylpropyldioxyl (HO-TME-O_2_^•^) and acetonyldioxyl peroxy radicals are formed, with HO-TME-O_2_^•^ arising from OH^•^ radicals adding to the TME double bond and then O_2_ adding to the carbon-centered radical.

**Figure 6:**
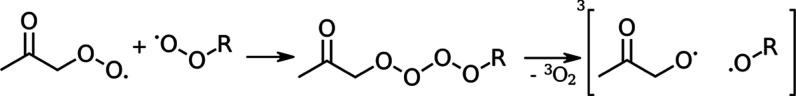
Formation of alkoxy radicals from two peroxy radicals going through a tetraoxide intermediate.

Acetonyldioxyl can react with other peroxy radicals going through a tetraoxide intermediate (RO_4_R′) which then splits off ^3^O_2_ and forms a triplet-state alkoxy–alkoxy complex, see Figure 6 for a schematic.^43,68^

**Figure 7:**
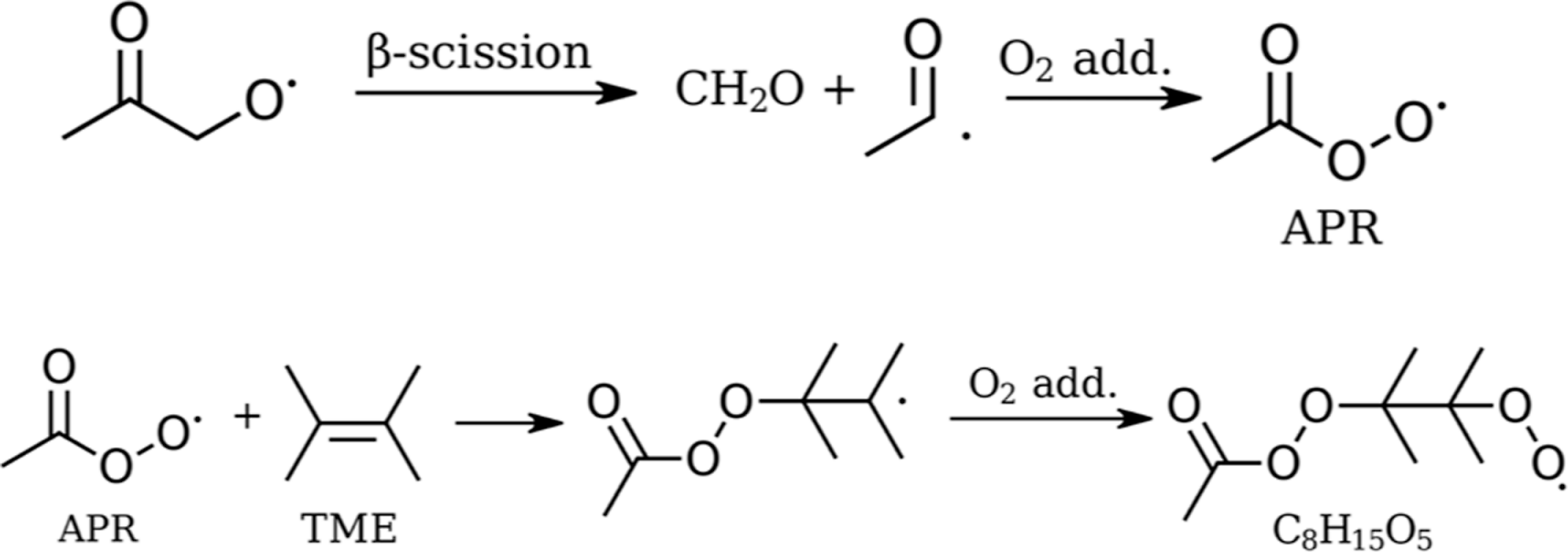
Unimolecular decomposition of acetonyl alkoxy radical through β-scission, generating formaldehyde and resulting in the acetyl peroxy radical. APR addition to TME resulting in a C_8_H_15_O_5_^•^ perester peroxy radical.

One of the possible products from this triplet-state complex is that it simply falls apart, resulting in free alkoxy radicals. The acetonyl alkoxy radical can undergo β-scission, according to the scheme in Figure 7.^69^ After O_2_ addition to the acyl radical the APR is formed.

In the experiments, the APR can add to the double bond in the TME according to the scheme in Figure 7. Theoretical calculations show that the reaction between APR and TME proceeds with a low energy barrier of 0.91 kcal/mol and a high reaction rate coefficient of 1.42 × 10^–15^ cm^3^ s^–1^. The concentrations of reagents used in the experiment are high enough to make the reaction possible. Moreover, the calculated reaction rate coefficient for TME + APR is similar order of magnitude (10^–15^ cm^3^ s^–1^) compared to reaction rate coefficients obtained for other unsaturated hydrocarbons in accretion reactions with APR (10^–14^–10^–16^ cm^3^ s^–1^, see Table 1).

**Figure 8:**
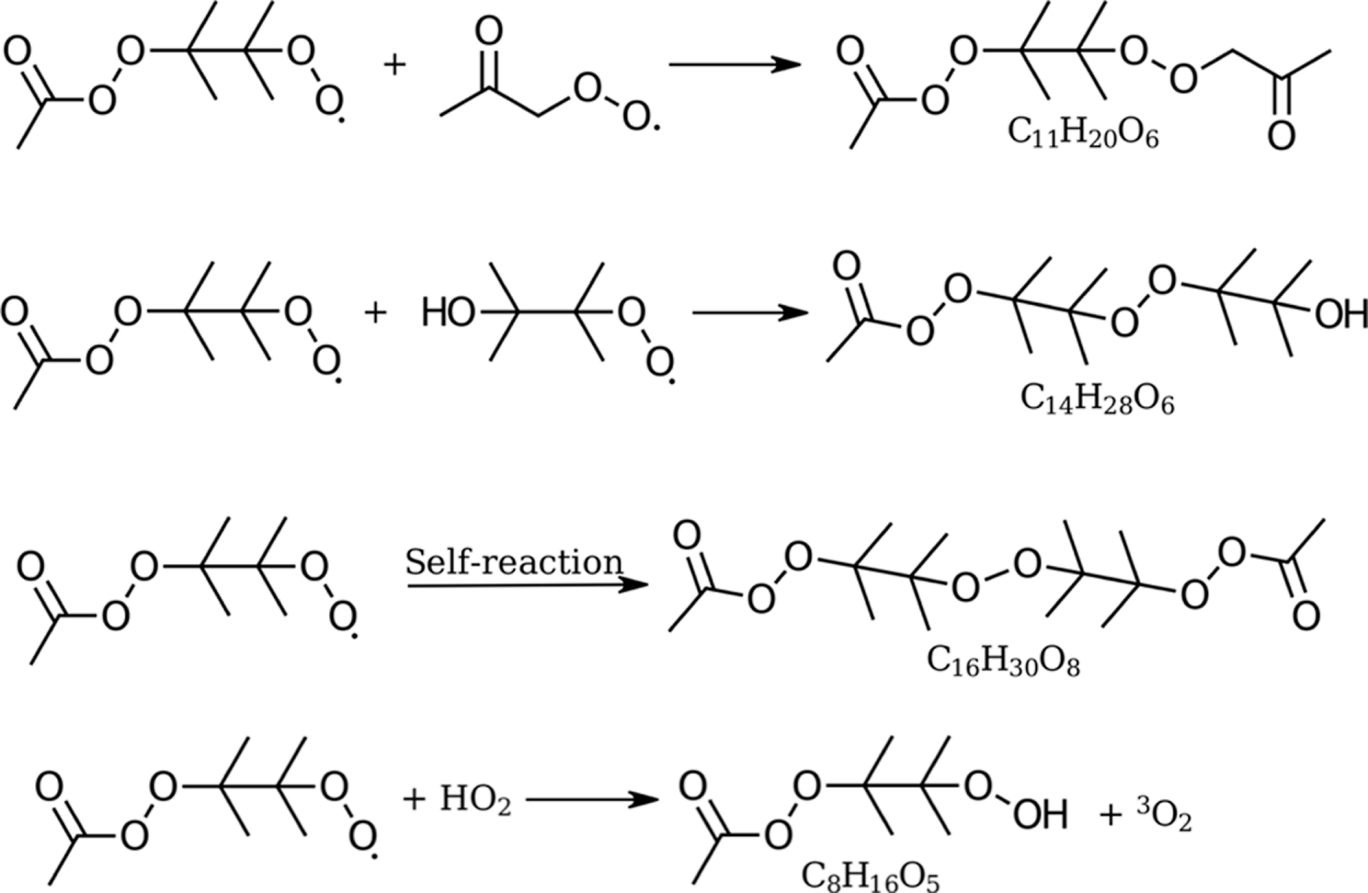
Reactions resulting in ROOR′ products involving the C_8_H_15_O_5_^•^ perester peroxy radical. All three products were observed in the flow reactor experiments. Termination reaction of the C_8_H_15_O_5_^•^ radical through hydrogen shift reaction with HO_2_^•^, resulting in C_8_H_16_O_5_ and ^3^O_2_.

In the flow reactor experiments, we could observe several closed-shell products that can be directly formed from C_8_H_15_O_5_^•^ perester peroxy radical termination reactions through reactions with other ROO^•^ in the flow reactor. Clear signals are observed at all TME injection rates, which are attributable to the ROO^•^ + R′OO^•^ reaction resulting in ROOR′ products, between C_8_H_15_O_5_^•^ and the two most abundant ROO^•^ radicals, acetonyldioxyl (C_3_H_5_O_3_^•^) and HO-TME-O_2_^•^ (C_6_H_13_O_3_^•^). The resulting masses correspond to sum formulas C_11_H_20_O_6_ and C_14_H_28_O_6_, respectively. A weak but clearly distinguishable signal which we attribute to the C_8_H_15_O_5_^•^ self-reaction forming the ROOR product (C_16_H_30_O_8_), can also be identified in the experiments with 1.0 and 2.0 μL/h TME injection rates. See Figure 8 for an overview of the reactions of C_8_H_15_O_5_^•^ perester peroxy radical leading to the experimentally observed ROOR′ products.

Furthermore, the mass corresponding to C_8_H_16_O_5_ was observed, which corresponds to a simple radical termination through the addition of hydrogen to the C_8_H_15_O_5_^•^ radical. The reaction partner is HO_2_^•^ which arises from HO_*x*_ chemistry, see Figure 8.

**Figure 9:**
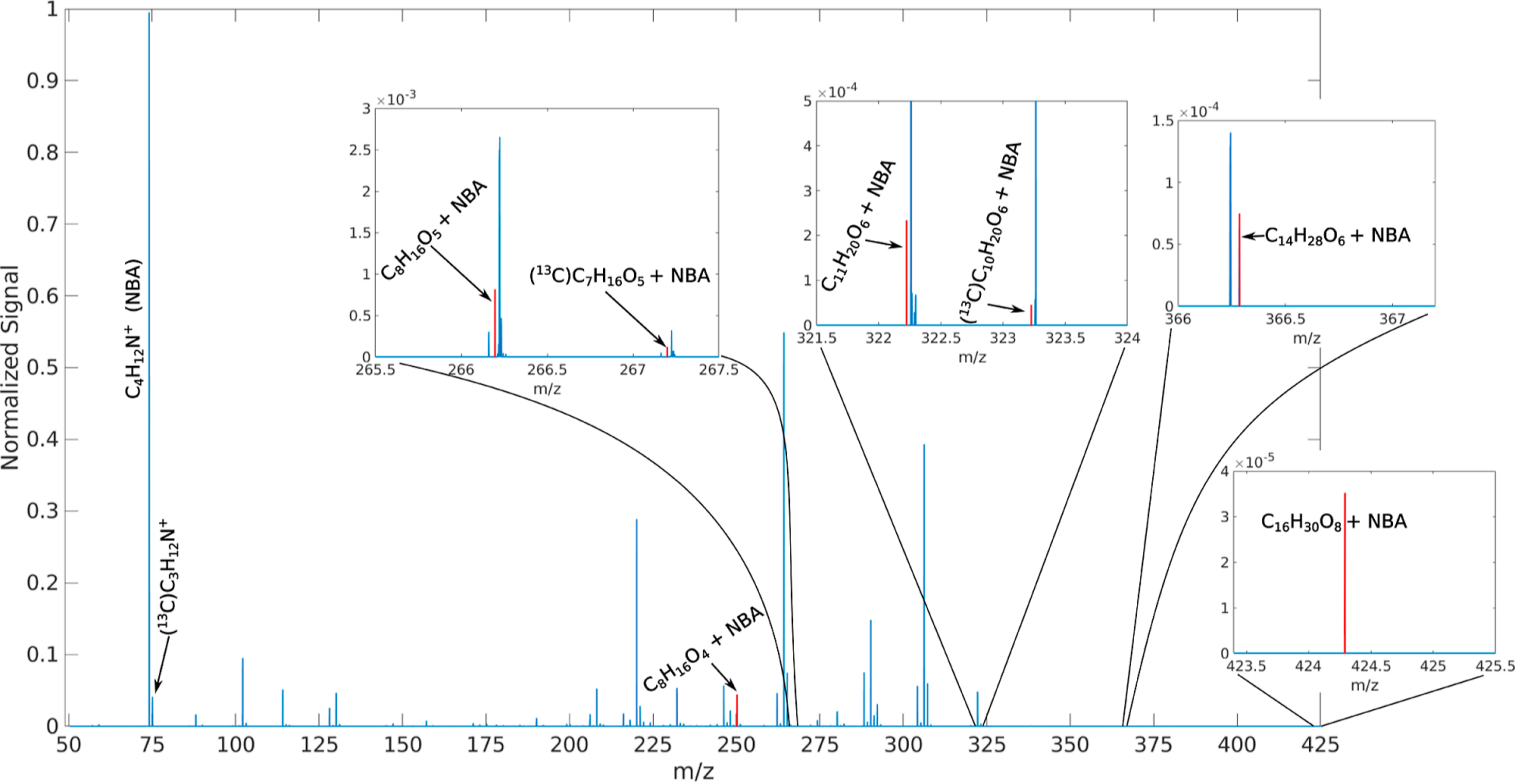
Mass spectrum from the 2.0 μL/h TME injection rate flow-reactor CIMS experiment. The APR related assigned peaks are highlighted in red. All other unlabeled significant peaks have been assigned but are not relevant to this work and therefore will not be discussed further.

A mass spectrum from the CIMS flow-reactor experiments is shown in Figure 9. The product signals from APR related chemistry are highlighted. The high resolving power and sensitivity of the CI-orbitrap employed for this work is why close lying signals can be confidently separately assigned. Each signal labeled in the figure was within 0.5 mDa of the calculated mass. Signals from ^13^C isotopologues were consistent with relative natural abundance to the ^12^C signals and had the expected *m*/*z* shift in the spectra, where they could be identified. For the weaker signals, from the C_14_ and C_16_ molecules, the main isotopologue signal was already so weak that the ^13^C isotopologue signal was not detectable.

**Figure 10:**
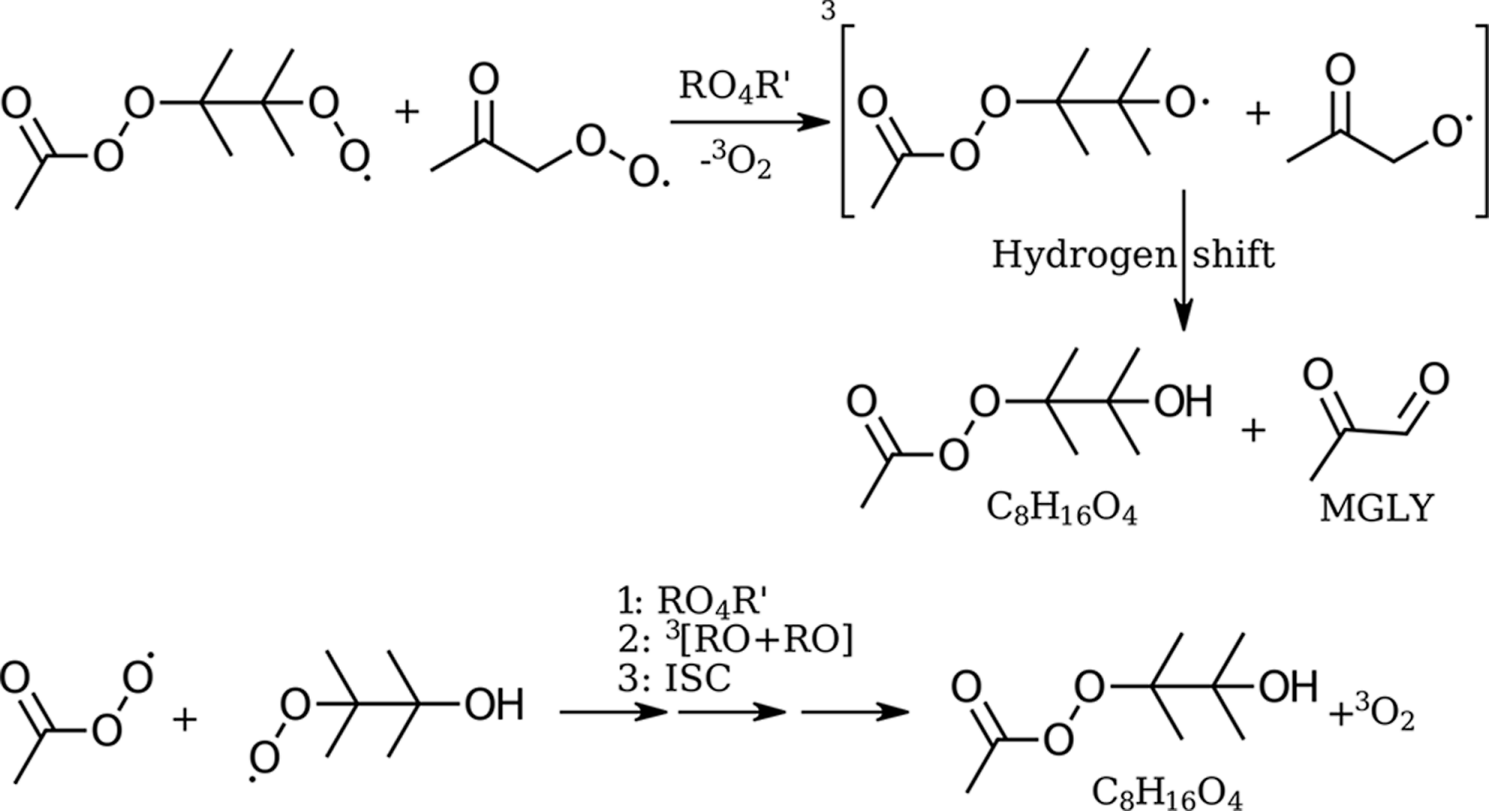
Different pathways to form the observed C_8_H_16_O_4_ mass. Either through a hydrogen shift reaction of the triplet alkoxy–alkoxy complex of C_8_H_15_O_5_^•^ + C_3_H_6_O_2_^•^ or from ROOR′ formation from the APR and the HO-TME-O_2_^•^ radical.

The C_8_H_15_O_5_^•^ perester peroxy radical can also participate in ROO^•^ + ROO^•^ chemistry like described in Figure 6, going through a tetroxide (RO_4_R′) step and forming an alkoxy radical (C_8_H_15_O_4_^•^) in a triplet complex with another alkoxy radical. One possible product from this triplet state alkoxy complex comes from a bimolecular hydrogen shift from the alkoxy carrying carbon to the radical oxygen on the other. For this to be possible, at least one of the two alkoxy radicals must have one or two hydrogens accessible on its alkoxy carrying carbon. In other words, if both alkoxy radicals are tertiary, then the hydrogen shift cannot occur. One of the two dominant peroxy radicals formed in the experiments, C_3_H_5_O_3_^•^ carries the peroxy on a primary carbon, and can thus lose a hydrogen from intermolecular hydrogen shift. The reaction is shown in Figure 10.

We note here that another way to form the C_8_H_16_O_4_ mass is through the ROOR′ reaction between APR and HO-TME-O_2_^•^. We expect the ROOR′ product from the reaction between APR and HO-TME-O_2_^•^ to dominate the C_8_H_16_O_4_ signal. This is based on the experimentally observed signal for the C_8_H_16_O_4_ mass, which is by far the most intense of all the masses we relate to the C_8_H_15_O_5_^•^ radical as shown in Figures 7, 8, and 10. Additionally, methylglyoxal is also observed experimentally, but that can arise from the hydrogen shift reaction in any triplet state alkoxy–alkoxy complex with the acetonyl alkoxy as one reaction partner. To elaborate, the ROOR′ product signals are generally the most intense signals we see in the experiments, with the ROOR′ product of the acetonyl peroxy radical self-reaction and the acetonyl peroxy reaction with HO-TME-O_2_^•^ being the two most dominant ones. Furthermore, it takes fewer reaction steps for APR to directly react with HO-TME-O_2_^•^ rather than first inserting into the TME double bond and then having the resulting ROO^•^ undergo further chemistry.

## 4 Atmospheric Implications

The results presented here demonstrate that APRs should be considered as potential oxidants of unsaturated hydrocarbons in the atmosphere. In the reaction with isoprene, all of the APRs yielded relatively low barriers and high reaction rate coefficients, up to 5 × 10^–15^ cm^3^ s^–1^. This implies that the isoprene + APR reaction might represent up to 0.1% of the total sink of isoprene (dominated by reaction with OH^•^ radical).

APRs also yielded low barriers for reactions with selected monoterpenes, particularly for limonene, for which the reaction rate coefficient is up to 10^–14^ cm^3^ s^–1^. Nonacyl peroxy radical exhibited uniformly low reactivity toward isoprene, and we believe that this low reactivity holds for other unsaturated hydrocarbons as well.

We further considered unimolecular H-shift pathways for the peroxy radical formed by the addition of O_2_ to the APR + isoprene product, with CH_3_C(O)OO^•^ chosen as the model APR. The fastest observed H-shift rate was 1.4 × 10^–3^ s^–1^, indicating that the predominant fate of this species is bimolecular rather than unimolecular reaction. These bimolecular reactions (with, for example, HO_2_^•^ and ROO^•^) would lead to various multifunctional products with 7 or more carbon atoms, which are not currently included in isoprene chemistry models. Analogous bimolecular reaction products, including likely ROOR′ recombination products with up to 16 carbon atoms, were experimentally observed to form in the ozonolysis of TME (a C6 alkene). Recent studies conducted by Dada et al. (2023) demonstrate that compounds with a minimum of 15 carbon atoms account for a large fraction of the oxygenated organic compounds with vapor pressures low enough to participate in the nucleation process.^70^ The present study shows that alkene + APR reactions, especially when coupled with ROO^•^ + ROO^•^ pathways, can lead to such accretion products even from relatively short-chain precursors such as TME. The reaction pathways reported here thus allow the formation of even larger and more functionalized products than previously believed. For larger systems, such as monoterpenes and sesquiterpenes, the APR + alkene + O_2_ products might also have competitive H-shift channels, opening up new autoxidation pathways.

## 5 Conclusions

In this study, we considered reactions of peroxy radicals containing various functional groups with unsaturated hydrocarbons. For the systems tested, only acyl peroxy radicals demonstrate sufficiently low energy barriers and high reaction rate coefficients to be potentially significant in the atmosphere. This was confirmed for isoprene, monoterpenes, and tetramethylethylene. Combined with the results by Pasik et al. (2024) concerning the isoprene reaction with aliphatic peroxy radicals,^29^ we are able to draw the following general conclusion. It appears that, for nonacyl ROO^•^ systems, reactions with unsaturated hydrocarbons are not feasible under atmospheric conditions and make a negligible contribution to atmospheric chemistry. For APRs, our theoretical and experimental results confirm that these reactions can occur under atmospheric conditions. While there are minor (on the order of a percent or less) channels in terms of the overall alkene sinks, the APR reactions lead to new and more functionalized (and hence lower-volatility) products. For example, APR chemistry combined with bimolecular recombination is observed to form C16 accretion products in the oxidation of the C6 alkene TME. This suggests that such pathways may need to be accounted for when modeling the formation of extremely low volatility compounds relevant to aerosol formation.

## Supporting Information Available

High-barrier perester peroxy radical pathways barrier heights; concerted and stepwise mechanism of HO_2_ loss; APT analysis; experimental details: calculation of TME initial concentrations (PDF)
Optimized structures and calculation output files of all relevant compounds that support the findings of the manuscript will be available in the Zenedo repository.
