# Correction to “Gas-Phase Oxidation of Atmospherically Relevant Unsaturated Hydrocarbons by Acyl Peroxy Radicals”

**DOI:** 10.1021/jacs.4c09636

**Published:** 2024-08-21

**Authors:** Dominika Pasik, Benjamin N. Frandsen, Melissa Meder, Siddharth Iyer, Theo Kurtén, Nanna Myllys

During further calculations on the reactions of unsaturated hydrocarbons with acyl peroxy radicals, we noticed an error in our previously published article “Gas-Phase Oxidation of Atmospherically Relevant Unsaturated Hydrocarbons by Acyl Peroxy Radicals”. In the article, we used the wrong structural conformer of the acetyl peroxy radical, which affects the reported energy barriers and reaction rate coefficients for reactions involving the CH_3_C(O)OO· and CH_3_CH_2_C(O)OO· radicals. This previously used conformer is structurally similar to global minimum conformers of other acyl peroxy radicals (APRs) as shown in Figure 1. However, in the case of the acetyl peroxy radical, the conformer is 0.5 kcal/mol higher in energy than the global minimum energy conformer.

**Figure 1:**
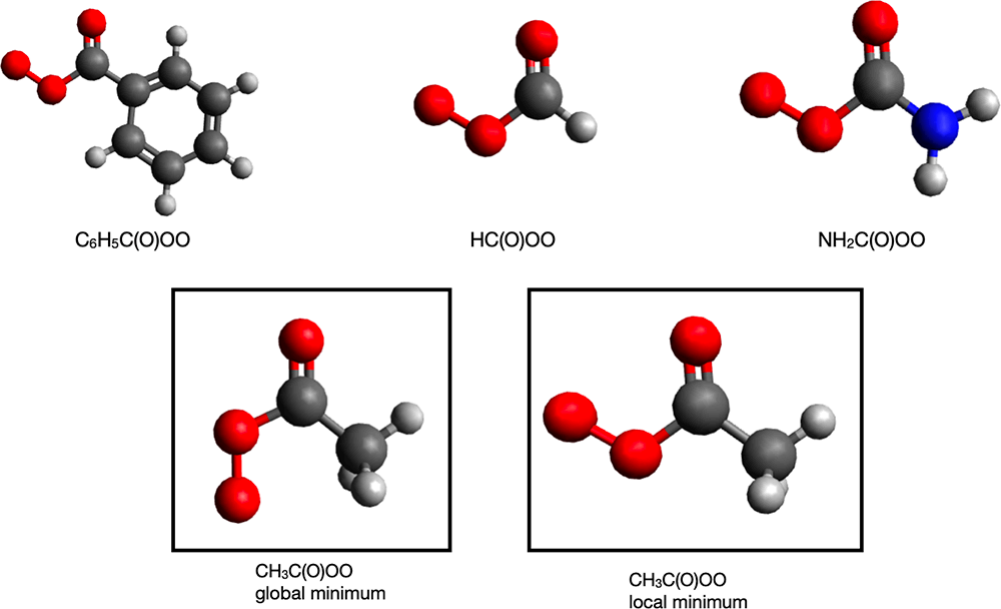
Global minimum conformer structures of various APRs (top). Global and local minimum conformer structures of CH_3_C(O)OO· radical (bottom). Color coding: carbon, gray; oxygen, red; nitrogen, blue; hydrogen, white.

In Table 1, we present the values for APR radical initiated oxidation of the studied unsaturated hydrocarbons by using the global minimum energy conformer.

**Table 1:** Zero-Point Energy Corrected Barrier Heights (Δ*E*^TS^ [kcal/mol]), Gibbs Free Energy Barriers (Δ*G*^TS^ [kcal/mol]), Bimolecular MC-TST Reaction Rate Coefficients at 298 K (*k*_bi_ [cm^3^s^–1^]), and Pseudo-First-Order Reaction Rates at 298 K (*k*_pseudo_[radical] [cm^–3^s^–1^]) for Studied Accretion Reactions of Monoterpenes and Toluene with Acetyl Peroxy Radical (Global Minimum) Calculated at the DLPNO-CCSD(T)/aug-cc-pVTZ//ωB97X-D/6-31+G* Level of Theory^a^

System	Δ*E*^TS^	Δ*G*^TS^	*k*_bi_	*k*_pseudo_[APR]	*k*_pseudo_[OH]
limonene-1	1.1	12.9	1.2 × 10^–15^	1.8 × 10^–7^	2.0 × 10^–4^
limonene-2	4.2	16.5	1.2 × 10^–18^		
limonene-3	2.8	14.7	6.4 × 10^–18^		
α-pinene-1	4.8	15.9	4.4 × 10^–19^	8.3 × 10^–10^	5.6 × 10^–5^
α-pinene-2	3.1	14.4	5.1 × 10^–18^		
β-pinene	2.4	13.8	2.7 × 10^–17^	4.1 × 10^–9^	8.8 × 10^–5^
tolunene-i	6.3	19.5	7.1 × 10^–22^	1.3 × 10^–12^	6.0 × 10^–6^
tolunene-o	6.1	17.8	6.6 × 10^–21^		
tolunene-m	7.8	20.3	1.1 × 10^–21^		
tolunene-p	7.7	19.7	4.5 × 10^–22^		
isoprene + CH_3_C(O)OO·	1.5	12.6	4.4 × 10^–16^	6.6 × 10^–8^	1.0 × 10^–4^
TME + CH_3_C(O)OO·	0.5	11.6	3.2 × 10^–15^	-	-
isoprene+ CH_3_CH_2_C(O)OO·	1.3	12.9	1.2 × 10^–16^	-	-

a Concentrations of reactants used to calculate reaction rates: [CH_3_C(O)OO·] = 1.5 × 10^8^ cm^–3^, [OH] = 1.0 × 10^6^ cm^–3^.

In light of the new data, the interpretation of results and atmospheric impact remains the same as we previously reported, which means the calculated reaction rate coefficients for reactions between APRs and investigated unsaturated hydrocarbons indicate minor but not necessarily negligible sinks in the atmosphere.

