# Supplementary material for: Gas-Phase Oxidation of Atmospherically Relevant Unsaturated Hydrocarbons by Acyl Peroxy Radicals
Source: J Am Chem Soc. 2024 May 7;146(19):13427–37. doi: 10.1021/jacs.4c02523 (PMC11389977; doi:10.1021/jacs.4c02523)
Supplement: Supplementary file 1 — ja4c02523_si_001.pdf [file ja4c02523_si_001.pdf]

Supporting information for Gas-Phase Oxidation of Atmospherically Relevant Unsaturated Hydrocarbons by Acyl Peroxy Radicals

Dominika Pasik, Benjamin N. Frandsen, Melissa Meder, Siddharth Iyer, Theo Kurtén, and Nanna Myllys

High-barrier perester peroxy radical pathways

Figure S1 shows rest of the studied unimolecular reactions for perester peroxy radical.

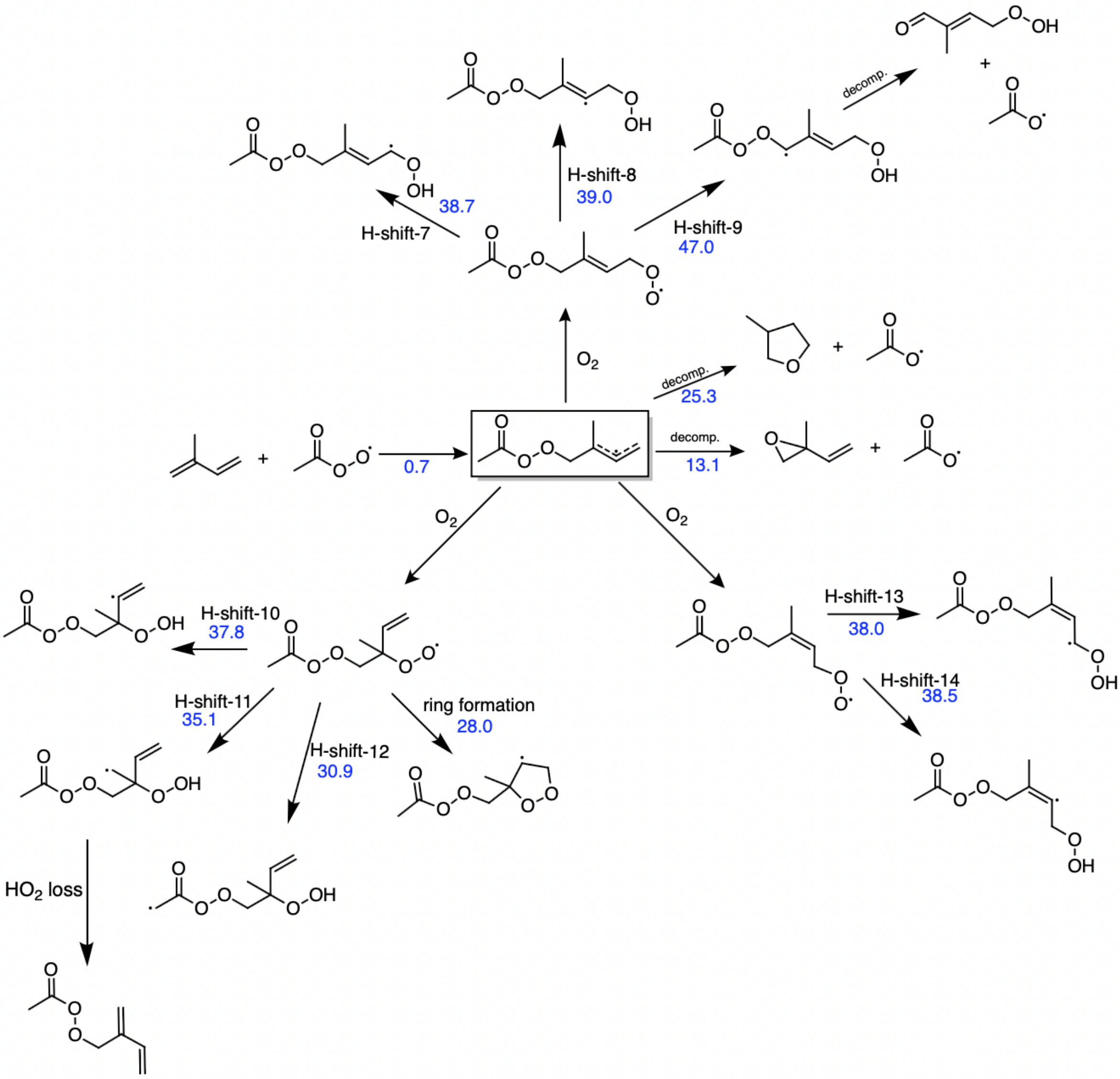

Figure S1: Studied pathway for accretion reaction between isoprene and acetyl peroxy radical and subsequent oxidation reaction channels.

Table S1 presents the zero-point energy corrected barrier heights for reactions shown in Figure S1. The reaction labeling is same in figure and table.

Table S1: Zero-point energy corrected barrier heights [kcal/mol] for alternative H-shift reactions calculated at the DLPNO-CCSD(T)/aug-cc-pVTZ//ωB97X-D/6-31+G* level of theory.

Reaction	Energy barrier
H-shift-7	38.6
H-shift-8	39.0
H-shift-9	47.0
H-shift-10	37.8
H-shift-11	35.1
H-shift-12	30.9
H-shift-13	38.0
H-shift-14	38.5
ring formation	28.0

Figure S2 illustrates the comparison between two mechanisms for HO_2 loss of the RO_2 radical.

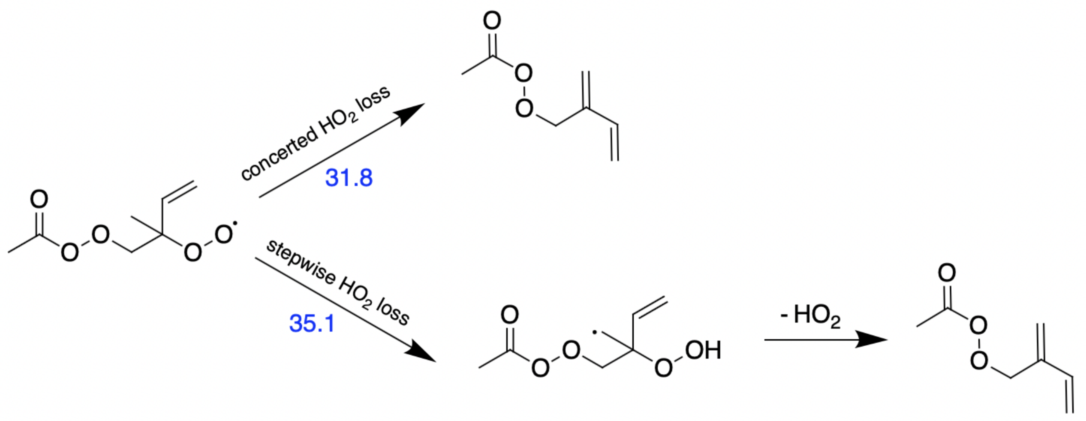

Figure S2: Direct comparison of the concerted (Figure 3) stepwise mechanisms (Figure S1) of HO_2 loss for the examined RO_2 radical.

Table S2: Zero-point energy corrected barrier heights and reaction energies (E_{TS}–E_{R} and E_{P}–E_{R} [kcal/mol]), Eckart tunneling factors (κ_t) and unimolecular MC-TST reaction rate coefficients at 298 K(k_{uni}, [s^{-1}]) for studied H-shift reactions calculated at the DLPNO-CCSD(T)/aug-cc-pVTZ//ωB97X-D/6-31+G* level of theory, number of unique conformers found in conformational sampling.

Reaction	E_{TS}–E_{R}	E_{P}–E_{R}	κ_t	k_{uni}	# of conformers
H-shift-1	27.2	1.7	5871	2.6×10^{-5}	22
H-shift-2	25.5	11.4	438	8.4×10^{-7}	5
H-shift-3	28.9	4.0	8	4.9×10^{-9}	16
H-shift-4	32.3	−27.7	3138	3.7×10^{-8}	1
H-shift-5	24.1	0.5	2088	1.4×10^{-3}	1
H-shift-6	26.9	13.4	510	2.5×10^{-7}	9

APT analysis

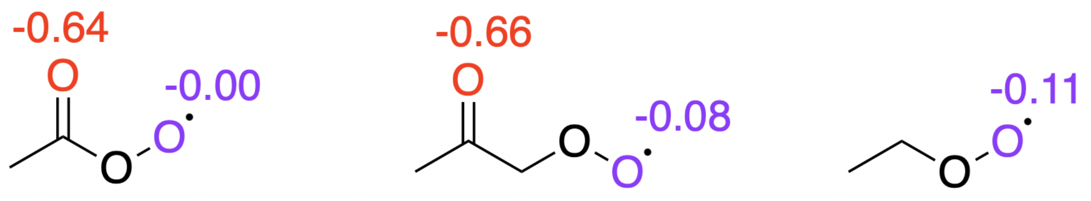

Figure S3: Atomic polar tensor (APT) analysis for studied radicals.

Figure S3 shows population analysis from atomic polar tensors for $CH_3C(O)OO\cdot$, $CH_3C(O)CH_2OO\cdot$ and $CH_3CH_2OO\cdot$ radicals.

Additional Experimental Details

Calculation of TME initial concentrations (prior to reactions taking place) in the flow reactor: Liquid TME in the syringe has a density of $\rho = 0.708$ g/cm^3 (25°C) according to vendor specifications (Sigma-Aldrich product number 220159) in other units $\rho = 7.08 \times 10^{-4}$ g/μL. The syringe pump injection rates used were 0.5, 1.0 and 2.0 μL/hr corresponding to 0.35, 0.70 and 1.4 mg/hr. The molecular weight of TME is M = 84.162 g/mol which means the injection rates correspond to 4.2, 8.4 and 16.78 μmol/hr. In the flow reactor, the total flow was 13.2±0.2 L/min or 792±12 L/hour. Dividing the injection rate with the total flow rates provides nominal TME concentrations of 5.3, 10.6 and 21.2 nmol/L. Using the ideal gas law p = (n/V)RT we obtain partial pressures for TME in the flow reactor corresponding to 1.3×10^{-2}, 2.6×10^{-2} and 5.3×10^{-2} Pa. Division of the TME partial pressures with standard atmospheric pressure $p_0 = 101325$ Pa results in the 128. 256 and 512 ppb listed TME concentrations in the article.
